# Photoreactions of cyclic sulfite esters: Evidence for diradical intermediates

**DOI:** 10.3762/bjoc.8.134

**Published:** 2012-07-30

**Authors:** Rick C White, Benny E Arney, Heiko Ihmels

**Affiliations:** 1 Department of Chemistry, Sam Houston State University, Huntsville, TX 77341, USA; 2 Department of Chemistry, Universität Siegen, Adolf-Reichwein-Str. 2, 57068 Siegen, Germany

**Keywords:** diradicals, mechanisms, photochemistry

## Abstract

The photochemistry of a phenyl and 1,2-diphenyl substituted sulfite ester is reported. The performance of photoreactions under relatively mild reaction conditions enables the detection of products that have not been observed in previous studies. It is concluded that, complementary to the initially proposed carbene intermediates, diradicals may also be considered.

## Introduction

Photoinduced ring-opening or ring-fragmentation processes constitute an important type of reaction in organic photochemistry and have been examined both from a mechanistic and a synthetic point of view [1–5]. Mechanistic studies have revealed information on reactive intermediates, solvent effects, and stereochemical outcomes, while synthetic work has pointed to novel approaches for preparing various molecules. For example, deazetation reactions in solvents of increasing viscosity were employed to determine whether the loss of nitrogen from cyclic azo compounds is stepwise or concerted [6]. The photoextrusion of molecular nitrogen was employed also synthetically in the photoreaction of a benzothiadiazole to give an antiaromatic derivative of benzothiirene [7]. In another case, Griffin proposed carbene intermediates in the photochemistry of vicinal diaryloxiranes based on the photochemical formation of methyl ethers in methanol solution [8]. Mechanistically, stereoselective oxygen scrambling was found in the photoextrusion of carbon dioxide from benzyl benzoate esters [9]. Furthermore, photodecarboxylation reactions in low-temperature matrices are the most efficient synthetic route to cyclobutadiene and its derivatives [10–12].

**Scheme 1:**
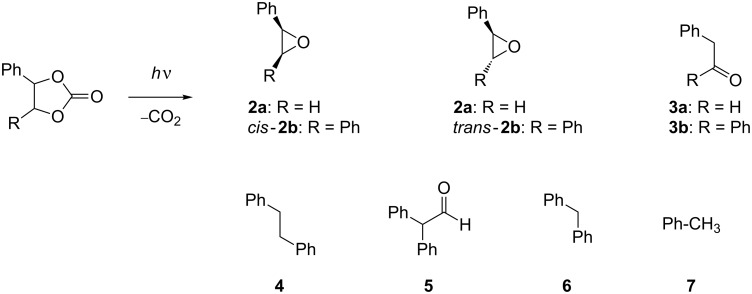
Photolysis of cyclic carbonate esters **1a** and **1b** in acetonitrile.

Mechanistic aspects of the photoinduced fragmentation of carbonate esters have been discussed. Thus, Griffin et al. reported that carbene intermediates are formed upon irradiation of cyclic carbonate esters such as **1b**, as evidenced by the interception with appropriate trapping reagents; whereas the formation of persistent primary photoproducts has not been observed [13]. In contrast, we have found that under significantly milder photochemical conditions (3 h versus 15 h), the cyclic carbonates **1a** and **1b** (Scheme 1) generate products derived from 1,3-diradical intermediates [14].

Interestingly, the photoinduced fragmentation reactions of nitrogen- and oxygen-containing functionalities have been studied intensively, whereas reports on photoextrusion reactions of sulfur-containing functionalities are rather rare. For instance, the photoextrusion of sulfur dioxide was carried out to facilitate the preparation of cyclophane derivatives [15]. The photochemistry of the corresponding cyclic sulfite esters is even less well known [16–18]. A report on the photochemistry of *meso*-hydrobenzoin sulfite undertaken about four decades ago stated that these compounds are also carbene precursors [16], in analogy to the results obtained with carbonate **1b**. To provide further insight into the mechanism of the photoinduced cleavage of the cyclic sulfites, we examined the styrene glycol sulfite (**8**) and the hydrobenzoin sulfite (**9**); and we herein demonstrate that diradical intermediates are generated during the photoextrusion processes.

## Results and Discussion

**Scheme 2:**
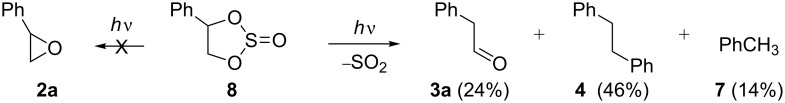
Photoreactivity of styrene glycol sulfite (**8**).

**Scheme 3:**
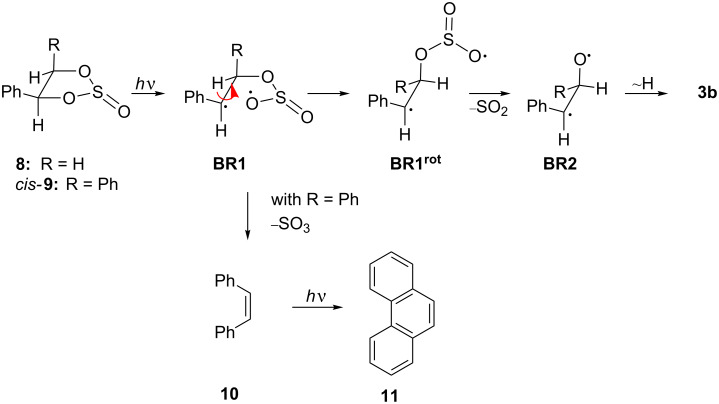
Photochemical pathway for photoextrusion of SO_2_ from cyclic sulfites.

Styrene glycol sulfite **8** was prepared from thionyl chloride and styrene glycol in the presence of triethylamine [19]. The irradiation of the cyclic sulfite **8** for 3 h in acetonitrile gave phenyl acetaldehyde (**3a**), bibenzyl (**4**) and toluene (**7**) in Scheme 2; however, in contrast to the results of the photoreaction of the cyclic carbonate ester **1a** under similar conditions, the oxirane **2a** was not detected as a reaction product. The lack of oxirane formation may be explained by initial cleavage of the C–O bond to give the biradical **BR1**, whose rotation about the C–C bond to give the relaxed biradical **BR1^rot^** is significantly faster than the loss of sulfur dioxide (Scheme 3). As a consequence, the elimination of SO_2_ results in a biradical **BR2** with a conformation that is unfavorable for oxirane formation, but enables efficient hydrogen migration to result in aldehyde **3a**. Theoretical calculations have confirmed that acetaldehyde derivatives may be formed upon rearrangement of the 1,3-oxyethandiyl [20–21] and that a subsequent decarbonylation results in an alkyl radical. The latter reaction yields bibenzyl (**4**) and toluene (**7**) in the photoreaction of **8**. It should be noted that the diradical **BR2** may be formed also upon photoinduced ring-opening reaction of an intermediate oxirane **2a**, which is not detectable in the reaction mixture because of its rapid secondary reaction. However, oxiranes have been observed as persistent intermediates under essentially identical conditions during photolysis of cyclic carbonates **1a** and **1b**, thus it is unlikely that they are formed to a significant extent in the photoreaction of **8**.

Similarly, we investigated the photochemistry of hydrobenzoin sulfite (**9**), which has been reported also to be a carbene precursor [16]. Because it has been shown in the case of cyclic carbonates that the application of milder reaction conditions enables the detection of reaction products that apparently decompose under harsher conditions, the original experimental conditions (*t* = 15 h) were changed. Thus irradiation of **9** in CH_3_CN for 120 min gave **5** and **6**, along with the so far undetected products **3b**, benzaldehyde (**12**), *cis*-stilbene (**10**) and phenanthrene (**11**). The same products were found after irradiation in cyclohexane. Such as in the case of the sulfite derivative **8**, the products **3b**, **5**, and **6** may be formed by photoinduced elimination of SO_2_ through a biradical intermediate (Scheme 3).

**Scheme 4:**
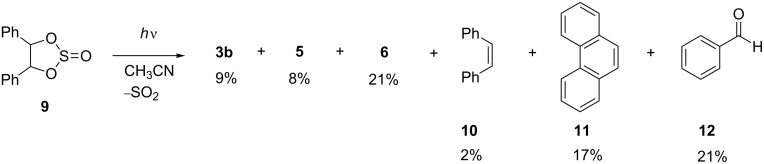
Photoreactivity of *meso*-hydrobenzoin sulfite (**9**).

Furthermore, no oxirane photoproduct could be detected, presumably due to the relatively slow elimination of SO_2_, thus resembling the photoreactivity of the derivative **8**. To be noted is the formation of **10** as a reaction intermediate**.** Although it has been observed that 4,4,5,5-tetraphenyl-1,3,2-dioxothiolan-2-one undergoes photoinduced fragmentation of SO_3_ to give 9,10-diphenylphenanthrene as a product through an initially formed tetraphenylethylene, the latter has not been detected [16]. The formation of **10** may be explained by the additional phenyl substituent, as compared to the cyclic sulfite **8**, which apparently facilitates the elimination of SO_3_ to give stilbene (**10**). Firstly, the phenyl substituent leads to a significant stabilization of the resulting benzyl radical as opposed to the formation of the oxylradical. Secondly, in the case of the *cis*-isomer of the cyclic sulfite **9** the cleavage of the C_benzyl_–O bond may be energetically favorable, because it releases significant steric strain by changing from sp^3^ to sp^2^ hybridzation (Scheme 3). The *cis*-stilbene (**10**) is subsequently transformed into phenanthrene (**11**) by photoinduced electrocyclization [22] as shown in Scheme 4.

## Conclusion

We have demonstrated that the performance of photoreactions of cyclic sulfites under relatively mild reaction conditions enables the detection of reaction products that have not been observed in previous studies. Notably, the analysis of the reaction products shows that the initially proposed reaction mechanism, which includes the formation of carbene intermediates, needs to be reconsidered. Specifically, the results presented herein suggest complementary reaction pathways through the formation of biradical intermediates.

## Experimental

**General remarks:** The NMR spectra were recorded on a Bruker Avance 400 (^1^H NMR: 400 MHz; ^13^C NMR: 100 MHz). ^1^H NMR chemical shifts are relative to tetramethylsilane (δ_TMS_ = 0.00 ppm). Melting points were determined on a Büchi 510K and are uncorrected. Phenyl benzyl ketone, diphenyl acetaldehyde, diphenylmethane, benzil, phenyl acetaldehyde, and bibenzyl were obtained commercially (Aldrich) and used as received.

**Styrene glycol sulfite** was prepared by adding thionyl chloride (2.5 g, 21 mmol) in diethyl ether (50 mL) dropwise to a solution of styrene glycol (2.8 g, 20 mmol) and triethylamine (4.6 g, 43 mmol) in ether (50 mL) with vigorous stirring. After being stirred for 4 h, the mixture was poured into water (100 mL). The ether layer was washed with water (4 × 50 mL) and dried (MgSO_4_). After removal of the solvent, the oil was distilled (148 °C at 2 mmHg) to give 2.29 g (60%) of pure styrene glycol sulfite as a mixture of syn/anti isomers (syn = phenyl groups and S=O bond on same side of ring); ^1^H NMR (400 MHz, CDCl_3_) δ 4.45 (dd, 1H), 4.25 (dd, 1H), 4.20 (dd 1H), 4.92, (dd, 1H), 5.38, (dd, 1H), 5.90 (dd, 1H) and 7.5 (5H).

***meso*-Hydrobenzoin sulfite.** Thionyl chloride (1.7 g, 14 mmol) in diethyl ether was added dropwise to a solution of *meso*-hydrobenzoin (2.1 g, 10 mmol) and triethyl amine, 2.5 g (25 mmol) in ether (100 mL). After being stirred for 3 h, the mixture was poured into water (100 mL), the ether layer was washed with water (4 × 50 mL) and dried (MgSO_4_), and then the ether was removed by rotary evaporation to give 2.24 g, (86%) of an equal amount of syn/anti isomers as colorless crystals; mp 109–110 °C. The ^1^H NMR spectrum showed two singlets at δ 5.8 (1H) and δ 6.1 (1H) in equal amounts as the syn and anti isomers.

**Photochemical reactions.** The sulfite esters (50 mg) were dissolved in acetonitrile (5 mL) in a quartz tube (13 × 100 mm), capped with a rubber septum, and then purged with argon for 45 min prior to irradiation. Photoreactions were carried out in a Rayonet Photochemical Reactor equipped with four RPR 2537 lamps and irradiated for three hours. The solvent was removed in vacuo, the reaction mixture was analysed by ^1^H NMR spectroscopy (400 MHz), and products were identified by comparison with literature data and with spectra of authentic samples. Specifically, after the NMR spectra had been recorded from the reaction mixture, authentic samples of reaction products were added, and the spectra were recorded again to verify the presence of reaction products. Yields for the reaction of **8** were calculated by adding redistilled *tert*-butyl alcohol and comparing integrations of the products with the methyl groups of the standard. Mass spectrometric data were recorded on photolysates immediately after reactions.

## Supporting Information

File 1 ^1^H NMR spectra of photolysates.
