# Supplementary material for: Photoreactions of cyclic sulfite esters: Evidence for diradical intermediates
Source: Beilstein J Org Chem. 2012 Jul 30;8:1208–12. doi: 10.3762/bjoc.8.134 (PMC3458739; doi:10.3762/bjoc.8.134)
Supplement: File 1 — 1H NMR spectra of photolysates. [file Beilstein_J_Org_Chem-08-1208-s001.pdf]

Supporting Information

for

Photoreactions of cyclic sulfite esters: Evidence for diradical intermediates

Rick C. White*[1], Benny E. Arney, Jr.[1] and Heiko Ihmels[2]

Address: [1]Department of Chemistry, Sam Houston State University, Huntsville, TX 77341, USA and [2]Department of Chemistry, Universität Siegen, Adolf-Reichwein-Str. 2, 57068 Siegen, Germany

Email: Rick C. White* - chm_rcw@shsu.du

* Corresponding author

^{1}H NMR spectra of photolysates

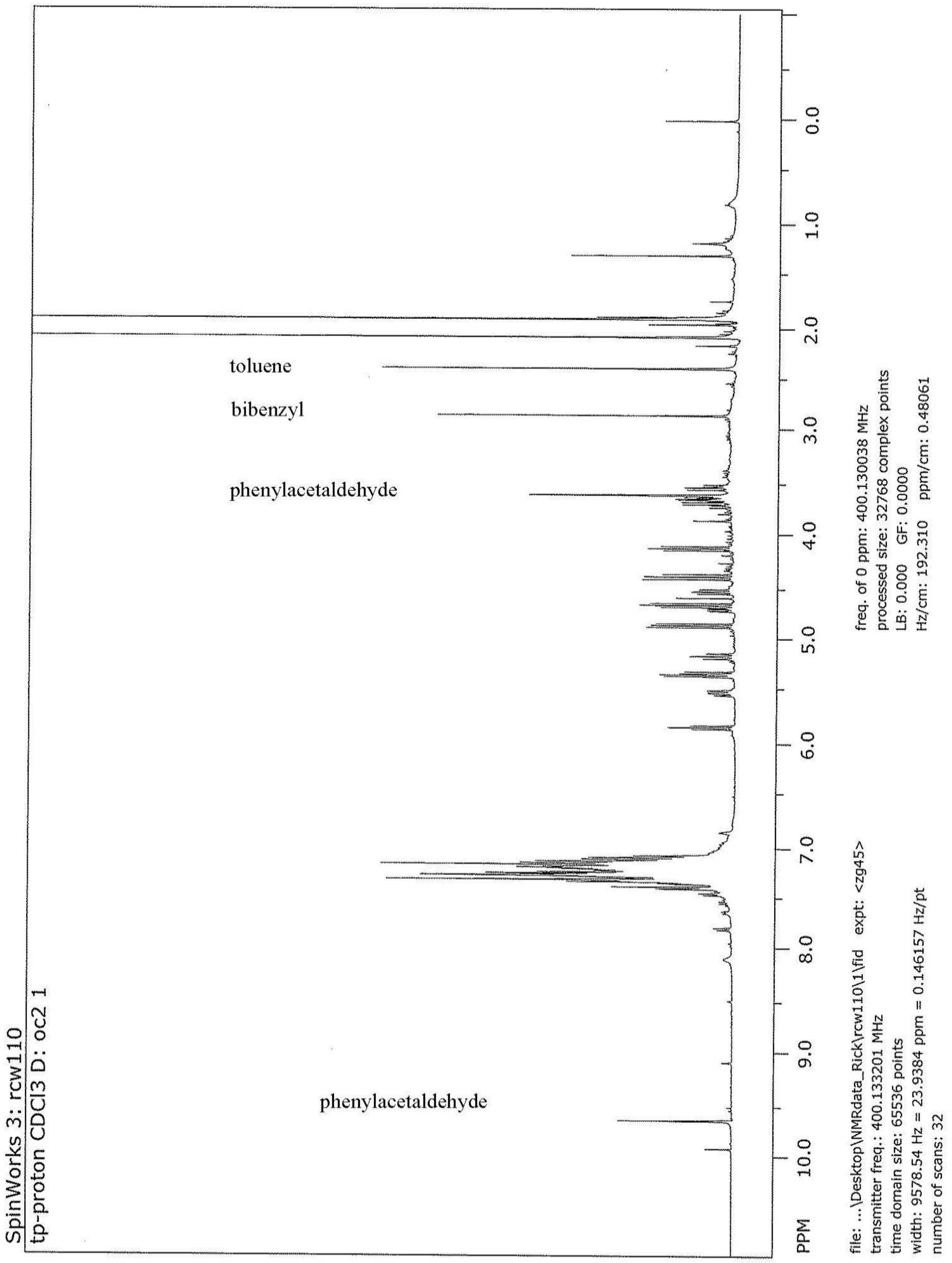

Figure S1: ^1H NMR spectrum of the reaction mixture after irradiation of styrene glycol sulfite (**8**).

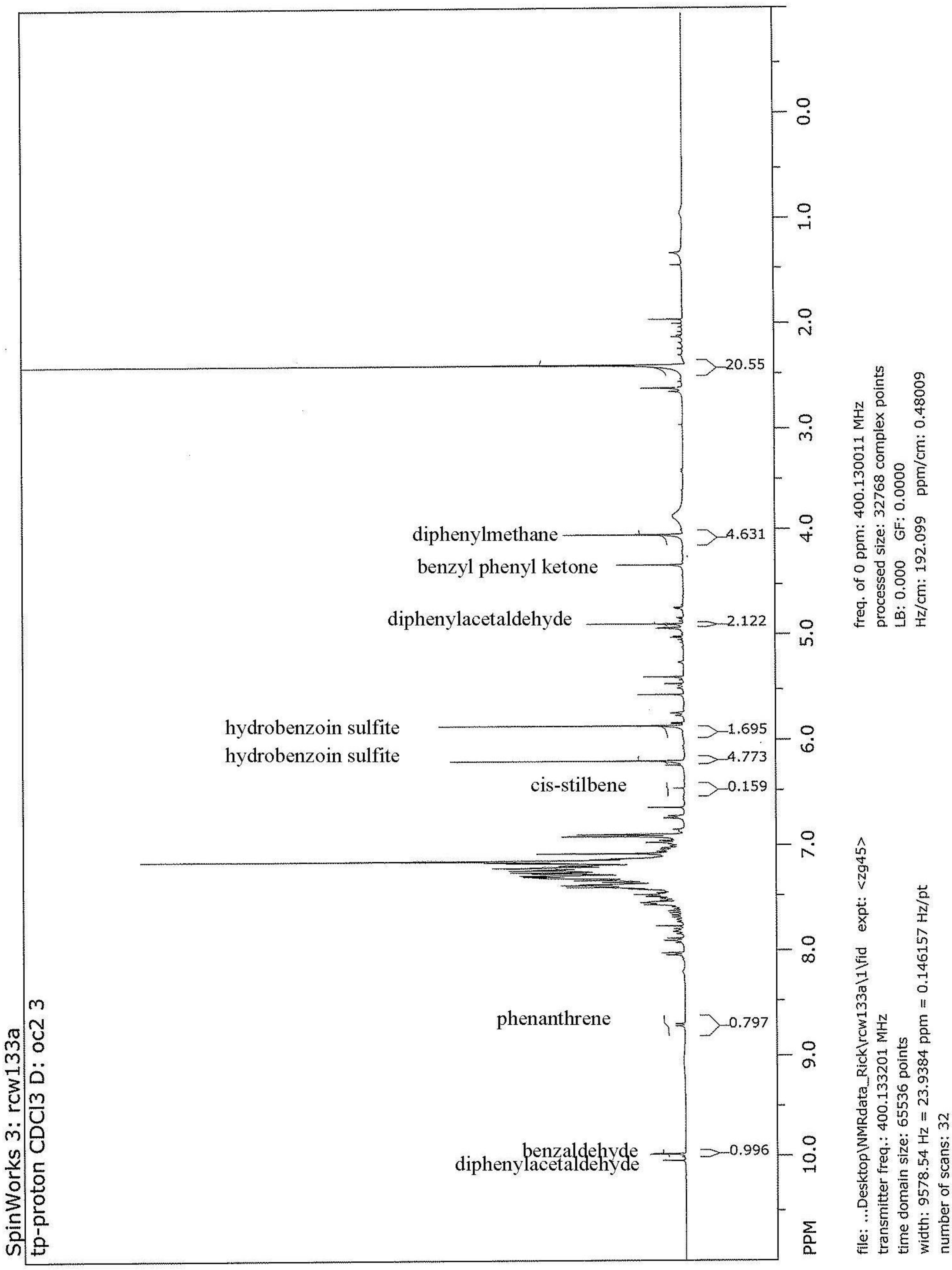

Figure S2: ^{1}H NMR spectrum of the reaction mixture after irradiation of hydrobenzoin sulfite (**9**).
